# Species rediscovery or lucky endemic? Looking for the supposed missing species *Leistus punctatissimus* through a biogeographer’s eye (Coleoptera, Carabidae)

**DOI:** 10.3897/zookeys.740.23495

**Published:** 2018-03-02

**Authors:** Pizzolotto Roberto, Brandmayr Pietro

**Affiliations:** 1 Dept. Biologia, Ecologia, Scienze della Terra, Università della Calabria, Italy

**Keywords:** climate change, endemism, ice ages, glacial periods, nunatak, mountain ecosystems, species rediscovery, Wallacean extinction

## Abstract

Is it correct to look for a supposedly missing species by focusing research at the type locality? A species can be declared extinct because for an unusual amount of time it has not been seen again; however, in the frame of the climate change it is likely that a supposedly missing species is a lucky survivor not seen because it was not searched for in the correct environment. We used the strictly endemic Leistus punctatissimus Breit, 1914 (Coleoptera, Carabidae) as the case study for testing the latter hypothesis vs. the type locality approach. On the basis of past unsuccessful searches in the Dolomites (a mountain range in the eastern Alps, Italy) driven by the type locality approach, a study area was selected where climate change may have exerted environmental constraints on endemic species. Five pitfall traps were used in each of seven sample sites, at an average altitude of 2600 m a.s.l., within a high altitude alpine plateau covered by scarce patchy vegetation. Leistus punctatissimus was rediscovered, far from its type locality, after one hundred years since its first collection. It was part of a group of species well adapted to the extreme ecological factors of the alpine environments above the vegetation line. Following a biogeographical approach (i.e., the biogeographer’s eye rather than the collector’s eye) it was possible to find an endemic species of the alpine ecological landscape in places from where it probably had never disappeared. The supposed refugial area was a nunatak during the last glacial period, where Leistus punctatissimus found suitable habitat conditions, and from where it alternated between downward and uphill changes in its distribution range after the last glacial period, under the effect of climate change. From such a perspective, it can be concluded that the type locality may be the wrong place to look for a supposedly extinct species.

## Introduction

When a species is “rediscovered” there are at least three reasons it was supposed to have disappeared (Scheffers et al. 2011): i) it disappeared as a consequence of a declaration of extinction by the researcher who rediscovered it; ii) when for any of several reasons it has been unseen for an unusually lengthy period of time; or iii) when it was collected for the second time only long after the collection of the type. A fourth reason has been proposed by Ladle et al. (2011), the lack of appropriate ecological and biogeographical knowledge because the species is only known from few specimens in museum collections. It is reasonable to suppose that the latter is the most probable explanation when dealing with relict species confined to high altitude environments.

*Leistus punctatissimus* Breit, 1914 is an ideal case study, because it is a relict species known for more than a hundred years on the basis of a female specimen lacking the abdomen (Breit 1914) and a male specimen dating back to the same epoch but described only recently (Assmann and Heine 1993) on the basis of which more complete knowledge of its morphology was obtained. From the date of the first collection on the Rolle Pass (Italy) by Anton Otto in 1902 (Breit 1914), the presence of the species has not been confirmed despite of extensive searches by Brandmayr and Zetto Brandmayr (1988), who focussed on a large altitudinal gradient of several environments from the bottom valleys to the talus slopes at high altitudes in the area surrounding Rolle Pass. A second research expedition was made along the same gradient in the years 2007–2012 (Pizzolotto et al. 2014, Pizzolotto et al. 2016) with the aims of detecting faunistic variations possibly occurring after the 1988 expedition and of sampling new habitats seeking for the presence of the beetle, but again, no trace of it was found.

The new research described in this paper was intended to determine if the distribution range of *L. punctatissimus* is other than what is arguable that based on the type locality (see Ladle et al. 2011), or if the distribution range has changed as a consequence of the climate change, as documented by the uphill shift (Pizzolotto et al. 2014) of two microthermal endemic species *Nebria germari* Heer, 1837 and *Trechus dolomitanus* Jeannel, 1931, once abundant in the area around Rolle Pass (Brandmayr and Zetto Brandmayr 1988).

## Methods

New environments were sampled in the years 2013–2014 following the hypothesis that the collection made by Anton Otto (Breit 1914) was the result of a fortuitous find, maybe at the limit of the distribution range of *L. punctatissimus*. We moved from the idea that climate change may have restricted the species’ geographic range much earlier, perhaps immediately after its discovery, according to the first well known temperature increase during the 1920s, as highlighted by the CRUTEM4 database (Jones et al. 2012).

The new sample sites had to show ecological features different from the sites sampled in the past, while at the same time they had to be characterised by features consistent with the presence of an endemic alpine species. Thus, we looked for the presumed missed species at higher altitude than in the past, in environments characterised by extreme ecological conditions, and covering an area large enough to ensure the long-term survival of viable populations also during ice age episodes.

**Figure 1.**
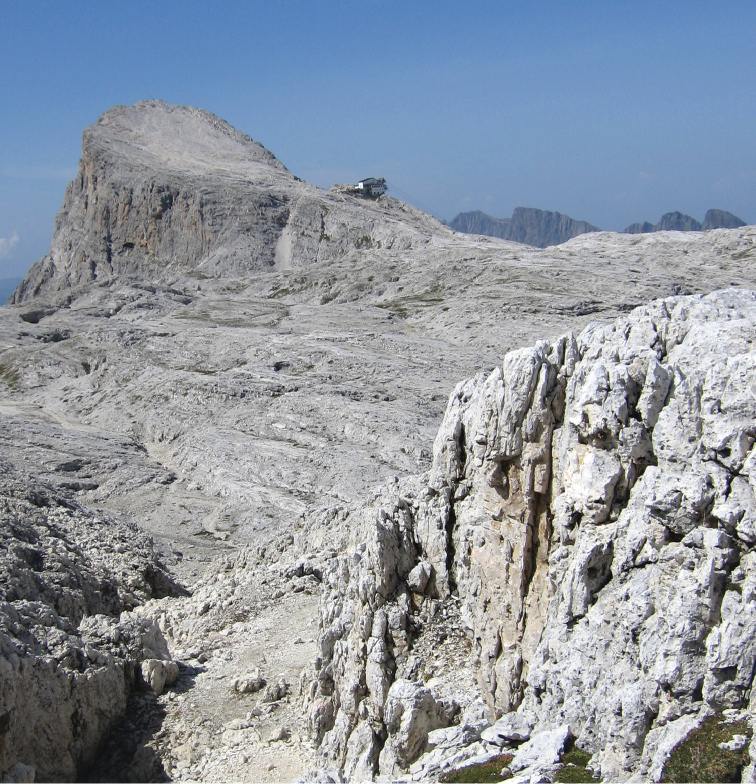
The study area “Altopiano delle Pale” was a karstic rock “desert” surrounded by several dolomite pinnacles, the one visible in this picture is the Rosetta Mount (2743 m a.s.l.) westward from the photographer. Behind it, to the right, the dark Lagorai mountain chain. The vegetation patchily colonised the study area exploiting the scarce presence of a thin lithosol. The “good” season usually lasts from the end of June to the beginning of September.

Our choice was directed by the topography of the mountains around the Rolle Pass and fell on the Altopiano delle Pale di S. Martino (Figure 1), a karstic rock “desert” surrounded by several dolomite pinnacles approximately 3,000 m high. Seven sample sites were selected having an average uphill shift of five hundred metres, at an average altitude of 2600m a.s.l., within a landscape typical of high altitude alpine environments where the vegetation cover is mostly composed of few pioneer species taking root on a thin lithosol patchily covering the rocks.

Five sites were selected in correspondence with the vegetation type mainly characterising the study area, which is classified in the eighth group (rocky habitats) of the NATURA 2000 (N2k) habitats (European Commission 2013), mainly a mosaic of limestone pavements habitat type (code 8240, N2k) and small patches of vegetation belonging to the calcareous and calcschist screes habitat type (code 8120, N2k). One site was selected on a talus slope covered with vegetation belonging to *Thlaspietea rotundifolii* Br.-Bl. in Br.-Bl. et Jenny 1926, code 8120, N2k, which characterises 10 % of the study area landscape. Another sampling site was selected on closed turfs (code 6170, N2k) within a small snowbed, which is a geomorphological element made by the seasonal accumulation of snow into small to large depressions; such landforms characterise 10 % of the area.

Five pitfall traps were placed in each site. A preservative mixture of wine vinegar oversaturated with table salt was used at the start of sampling and each time after the traps were emptied. Due to the low temperatures it was possible to empty the traps two or three times during July and September of each year.

## Results

### Ground beetle samples

In the seven sample sites twelve species of ground beetles were found, mostly endemic of the Dolomites (n = 7; 58 %) and with short wings (i.e., brachypterous, n = 8; 67 %). Among these *L. punctatissimus* was collected for the first time after more than one hundred years since its first collection and description. Three specimens were found in the pitfall traps, two females and one male, all deteriorated due to bad weather conditions during trapping.

Among the sampled taxa, two species have wider distributions, *Bembidion glacialis* (Heer, 1837) and *Pterostichus morio* (Duftschmid, 1812), known from the Alps, the northern part of the Apennines, and Carpathian Mountains, while two species, *Notiophilus biguttatus* (Fabricius, 1779) and *Amara erratica* (Duftschmid, 1812), have European and Palaeartic distribution range, respectively.

The species most frequently found in the study area were *Nebria germari* (100% of the sampled sites), *N. diaphana* (K. Daniel & J. Daniel, 1890), *Carabus bertolinii* Kraatz, 1878, *C. creutzeri* Fabricius, 1801 (all in 86 % of the sites; n = 6), and *Trechus dolomitanus* (n = 4; 57 % sites).

The highest diversity was found in the snowbed environment, where 83 % of the sampled species (n = 10) were collected, while the talus slope environment harbours 50 % of all species recorded. In all the sites except one, the patchy pioneer vegetation site, species richness was less than the 50 % of the sampled species. All the species collected except *A. erratica* have zoophagous diet.

### Temperature trends

**Figure 2.**
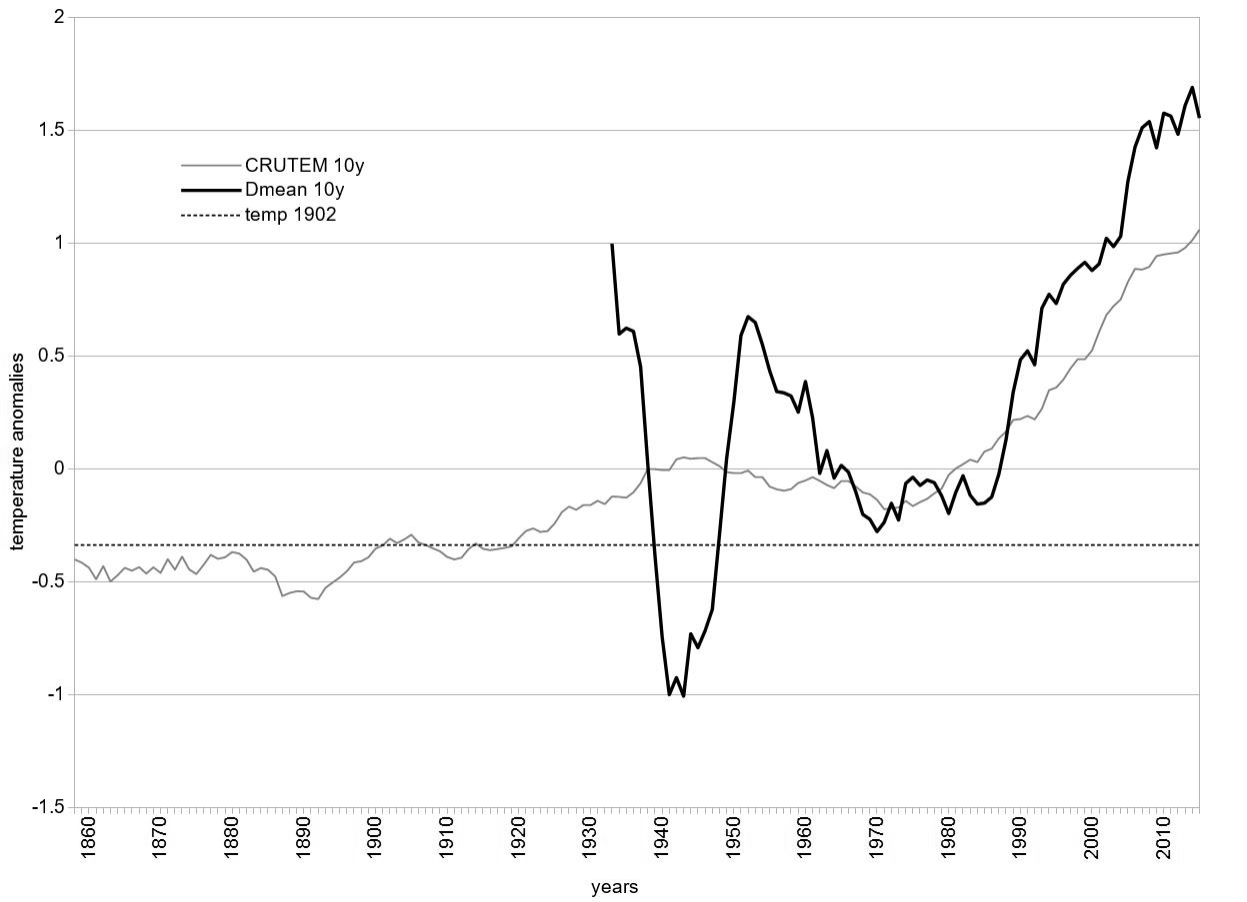
Trends of global and local ten years running average temperature anomalies. The local (Rolle Pass) temperature trend (bold black line, based on the Meteotrentino database, see Data Accessibility) consistently with the global temperature trend (based on CRUTEM4 database) is expressed as temperature anomalies from the base period 1961–90 (see Jones et al. 2012). The horizontal dotted line shows the average global temperature anomaly in 1902, after this year the global trend cross the line downwards only one time. During the last 30 years, global and local trends are consistent with the climate change hypothesis.

The average temperature trend at the Rolle Pass is given in Figure 2, where it is compared with the northern hemisphere temperature increase during the last century (CRUTEM4 database), and with the CRUTEM4 average of 1902. Especially in the last four decades, the average values show a strong increase, but a preliminary warming of the area may have occurred even before this, beginning at least during the 1930s when measurements first began at the Rolle Pass, as is evident in Figure 2.

### Palaeogeographic reconstruction

**Figure 3.**
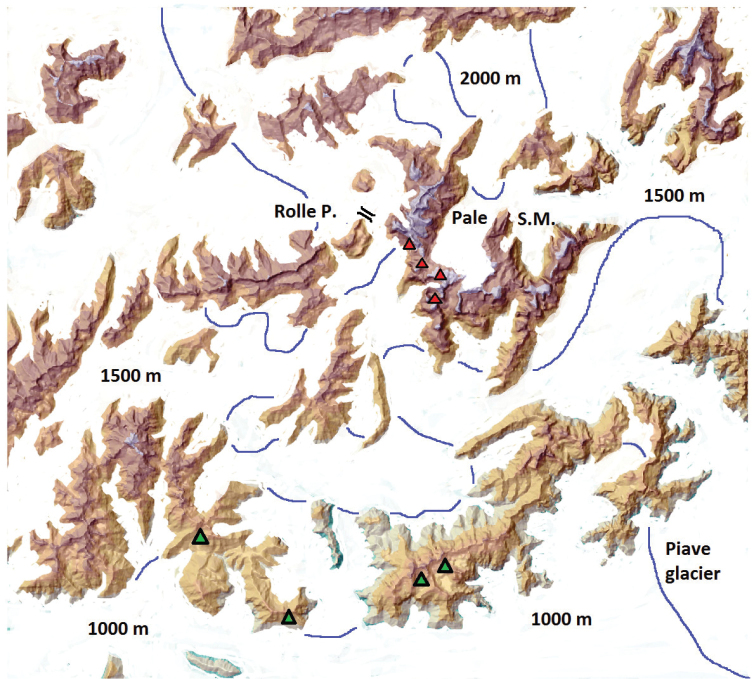
The possible distribution of *Leistus punctatissimus* during the last ice age maximum, (red triangles). Green triangles: northern “sentinels” of *Duvalius breiti*, a relict of the “Massifs de refuge”. The blue lines indicate the approximate height of the alpine “inlandsis” of the Dolomites around the Rolle Pass. Reconstructed after the map of Pellegrini et al., 2004. The Pale di San Martino massif represents one of the largest nunatak area near the Rolle Pass.

**Figure 4.**
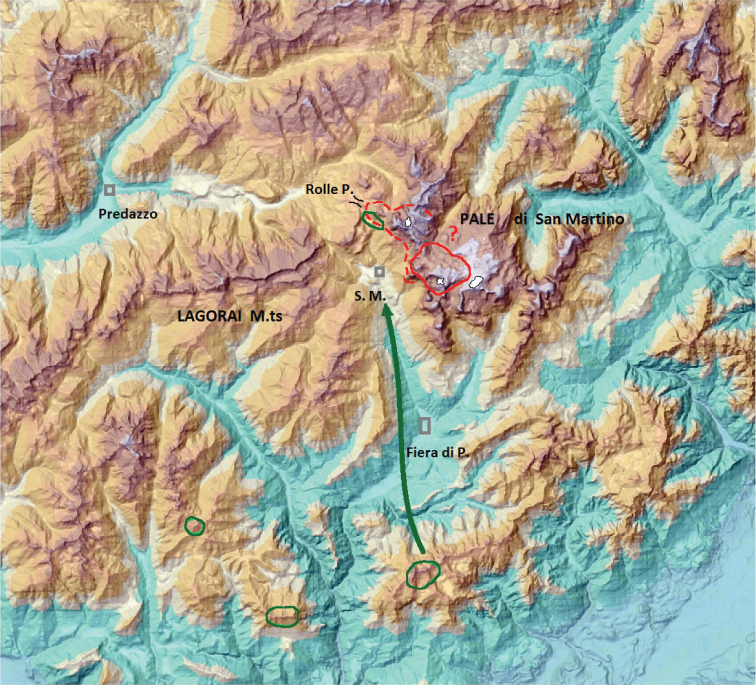
Present distribution area of *Leistus punctatissimus* (continuous red line) and putative western boundaries of the same around year 1900 (broken red line). The eastern boundaries of the area remain uncertain. The glaciers as present today are represented by white spots with black borders. Green areas: presently known distribution of *Duvalius breiti*. The green arrow indicates the possible re-immigration pathway of this species towards Rolle Pass. S. M.: San Martino di Castrozza.

The map presented in Figure 3 shows the presumable distribution of ice cover during the last glacial age (Würmian) and the possible “overwintering” sites of *L. punctatissimus* on the Pale di S. Martino high plateau. In Figure 4 the same area is redrawn as it is during the present time, where the remaining small glaciers are reduced to nearly invisible spots. The distribution of the beetle was reconstructed on the basis of Pizzolotto et al. (2014), who found in the same area an uphill shift of the distribution range of several species as a consequence of the climate change. The described uphill shift can be used to reconstruct the distribution ranges also of former periods as in Figure 4, where the putative distribution of *L. punctatissimus* is restricted to the plateau, while it was wider than now at the beginning of the last century. .

## Discussion

The paths rising to the Dolomites plateaus have a long history, being visited by tourists since the mid-nineteenth century (Gilbert and Churchill 1864). However, there is no certainty of knowing whether or indeed how many entomologists went so far. It is a matter of speculation whether Anton Otto in 1902 had preferred a convenient walk around the Pass, where he found a pair of specimens of *Leistus punctatissimus*, or whether he intentionally went climbing on the surrounding mountains.

After thorough searches following the footsteps of Anton Otto, which lead to no further findings of *L. punctatissimus*, it was hypothesised that the core of the distribution range could have been linked to the environmental conditions shaped by the effect of the ice age.

From a biogeographical point of view, a species narrowly distributed inside an alpine landscape, above the tree line of a southern European mountain chain, is seen as the product of evolution and area restriction moulded by the ice ages. Consequently, the species should be looked for within areas possibly free from ice cover, corresponding to a nunatak or a peripheral glacial refugium (Holderegger and Thiel-Egenter 2009). Conversely, following the “collector’s eye” the same species should be looked for at or near the type locality. Indeed *L. punctatissimus* was found as a very rare species among a group of twelve species in the Dolomites, most of these stenoendemic and brachypterous. Among these, worthy of a mention are *Nebria germari* and *N. diaphana*, which inhabit high altitude environments colonised by small patches of vegetation in the Dolomites (Brandmayr and Zetto Brandmayr 1988, Pizzolotto et al. 2014). From a general point of view, based on biogeographical and autoecological features of our sampled carabid fauna, the Pale di S. Martino plateau is recognised as a relevant nunatak in the eastern Alps at least during the last glaciation. At the same time, the Rolle Pass played the role of a northern threshold in the pathway of distribution for the short-distance re-immigrating species, arriving from the “massifs de refuge” of the southern Alps (see Holdhaus 1954).

Assmann (1997) suggested that the absence of specimens of *L. punctatissimus* from pitfall trap samples taken in the Rolle Pass area was simply due to successful avoidance of the traps by members of this species. Even if Perrault (1982) placed *L. punctatissimus* in synonymy with *L. ovipennis* Chaudoir, 1867, the latter does not occur at Rolle Pass, so that no taxonomic issue could explain the disappearance of *L. punctatissimus*. However Assmann and Heine (1993) provided morphological data supporting these two as separate species, an opinion shared also by Farkac (2005).

It is likely that *L. punctatissimus* is another case of “Wallacean extinction” (Ladle and Jepson 2008), which refers to a species poorly documented because the survey of its populations has been discouraged by the severe environmental conditions researchers had to face (see also Ladle et al. 2011). Our results highlight that, rather than focusing on the type locality, it is better to derive the possible habitat preference of the supposedly missed species on the basis of its biogeographical history, and then to derive the possible bioclimatic zone which is expected to harbour the target species. It appears that a biogeographical approach is more informative than a purely geographical one in these cases. This situation is comparable to the history of a relict species of the “massifs de refuge” studied by Holdhaus (1954), the microphthalmic carabid beetle *Duvalius breiti* (Ganglbauer, 1900), found at the Rolle Pass by Brandmayr and Zetto Brandmayr (1988).

The question that remains controversial is whether our first searches might have been misled by the consequences of climate change. This would imply an early contraction of the species’ distribution range, perhaps immediately after its discovery according to global warming data, which after the beginning of the 20^th^ century shows a clear upward trend (Figure 2, see the 1920’s temperature line). However the species is strictly stenoendemic, so that it responds to local temperature trends, and the local temperature trend shows that a time of climate cooling occurred between the discovery of *L. punctatissimus* and the 1980s expeditions (Figure 2, during the 1940s). It would be possible to hypothesise that during that cooling time, the species could have expanded rather than contracted its distribution range.

A reasonable explanation is that this cooling time was too short for the species to respond. Expanding this reasoning based on the theories of Vincent et al. (2005), one could hypothesise that the shrinking of the *L. punctatissimus* distribution range began at the end of the Little Ice Age when the temperature factor became more critical than the lack of snow precipitations.

Again, seeking the species on the basis of its biogeographical features provided the searching clue consistent with the actual distribution of the species, which was rediscovered within an environment of approximately 2600 metres altitude, where the favourable season for insect activity is a short cold spring (compared with the bottom valley climate), and the vegetation is at the upper limit of survival. These are environmental features generally in line with the autoecological features of an endemic species of the Alps, which lead us to consider the initial discovery by Anton Otto as a fortuitous finding, while our first failures were the result of an erroneous “geographically driven” research.

Another question requiring an answer is about the origin of the species. Following Habel et al. (2010), we can hypothesise that *L. punctatissimus* is a relict species that evolved, after the recolonization from the Pontic-Mediterranean glacial refugia, from a group of *Leistus* which lead to the allopatric differentiation of eight species (Assmann and Heine 1993). The speciation was a consequence either of the Holdaus Line effect (Drees et al. 2010), or of the disjunction of a population along the northward recolonising route. Assmann and Heine (1993) outlined that the distribution range of *L. punctatissimus* was known to be very small, only the region around the Rolle Pass, an area which had been covered by ice during the last glaciation. On this basis they hypothesised that the species should has come from a refugial landscape like, for instance, *Duvalius breiti*, and it should be considered as a short-distance re-immigrant (Holdhaus 1954).

As it is not easy to reconstruct the kinship among *L. punctatissimus* and those eight species, and given the very narrow distribution range of each, it could be hypothesised that *L. punctatissimus* is a species which evolved *in situ*. This opens new perspectives for biogeographical studies of the Oriental Dolomites, because these mountains may have played the role of nunatak, while the same is not arguable from a mere geographical or geomorphological analysis.

The survival on nunataks is likely for other ground beetles also. This is especially true for Nebriine species to which also *Leistus punctatissimus* belongs (e.g. *Oreonebria atrata*: see Holdhaus 1954; *Oreonebria bremii* and *O. bluemlisalpicola*: see distribution maps in Szallies and Huber 2014). Moreover, several other ground beetle species survived on permafrost ground conditions the last glacial period in Central Europe as revealed by strikingly differentiated populations (e.g. Homburg et al. 2013, Drees et al. 2016). Therefore, it seems to be likely that many ground beetle species are able to survive under low temperature conditions than previously thought (cf. discussions in Drees et al. 2016).

Permanent monitoring of the study area will inform us if the population of *L. punctatissimus* is in a stable or in a contracting state in light of climate change, while a daring entomological exploration of the peaks crowning the S. Martino valley will led to the reconstruction of its actual distribution range, telling us if the species evolved *in situ* or if it re-immigrated from southern glacial refugia.

## Conclusions

*Leistus punctatissimus* is likely a nunatak survivor that “overwintered” on the extremely large nunatak of the Pale di San Martino and its karstic alpine upland. In the postglacial period it alternated between downwards and uphill changes of the distribution range, colonising the southern slope of the Rolle Pass and its large debris and stone fields during the postglacial retreat of the ice.

The last of such “area pulses” happened during the small ice age of the eighteenth and nineteenth centuries. During this epoch, *L. punctatissimus* and *Duvalius breiti* populations lived together in the erosion landscapes and debris channels of the southern slopes of the Rolle Pass. Shortly thereafter the extremely microthermophilous *Leistus* species was forced to leave the open habitats around the Rolle treeline, whereas the more thermophilous (and hygrophilous) *Duvalius* species survived in humid erosion channels and along, stone-rich runlets.

Last, but not least, in the new perspective of global warming: it should be kept in mind that a type locality of the past century could not be the appropriate collecting target.

## Data accessibility

All topographic and environmental GIS layers generated for this study are available from the cartographic databases of the Trento Province (http://www.territorio.provincia.tn.it/portal/server.pt/community/s_i_a_t/255/s_i_a_t/18995), and the Veneto Region (http://idt.regione.veneto.it/app/metacatalog/).

All climatic data used for this study are available from the CRUTEM4 database (https://www.metoffice.gov.uk/hadobs/crutem4/), and the Meteotrentino, i.e. the Trento Province database for climatic data (http://www.meteotrentino.it/dati-meteo/stazioni/elenco-staz-hydstra.aspx?id=168).

## References

[B1] Assmann T (1997) A new species of *Leistus* Frölich from the Picos de Europa, Cantabrian mountains, Spain (Coleoptera: Carabidae). Koleopterologische Rundschau 67: 1–4.

[B2] Assmann T Heine S (1993) Die *Leistus*-Arten der *Oreobius*-Gruppe: Systematik, Taxonomie und Verbreitung (Coleoptera, Carabidae: Nebriinae). Mitteilungen aus der Entomologischen Gesellschaft Basel 43: 42–68.

[B3] Brandmayr P Zetto Brandmayr T (1988) Comunità a coleotteri carabidi delle Dolomiti Sudorientali e delle Prealpi Carniche. Studi Trentini di Scienze Naturali, Acta Biologica 64: 125–250.

[B4] Breit J (1914) Beschreibung zwölf neuer palaearktischer Coleopteren Formen aus der Familie Carabidae. Coleopterologische Rundschau 10/11: 155–170.

[B5] Drees C Matern A von Oheimb G Reimann T Assmann T (2010) Multiple glacial refuges of unwinged ground beetles in Europe: molecular data support classical phylogeographic models. In: Habel JC Assmann T (Eds) Relict Species: Phylogeography and Conservation Biology. Springer-Verlag, Berlin Heidelberg, 199–215. https://doi.org/10.1007/978-3-540-92160-8_11

[B6] Drees C Husemann M Homburg K Brandt P Dieker P Habel JC von Wehrden H Zumstein P Assmann T (2016) Molecular analyses and species distribution models indicate cryptic northern mountain refugia for a forestdwelling ground beetle. Journal of Biogeography 43: 2223–2236. https://doi.org/10.1111/jbi.12828

[B7] European Commission DG Environment (2013) Interpretation Manual of European Union Habitats. http://ec.europa.eu/environment/nature/legislation/habitatsdirective/docs/Int_Manual_EU28.pdf

[B8] Farkac J (2005) Systematic Outline and Geographic Distribution of Species of the Genus *Leistus* Frölich, 1799 (Coleoptera: Carabidae: Nebriini). Studies and reports of District Museum Prague-East Taxonomical Series 1: 43–67.

[B9] Gilbert J Churchill G (1864) The Dolomites Mountains. Longman. 2017 reprint by Kalpaz, Delhi, 576 pp.

[B10] Habel J Drees C Schmitt T Assmann T (2010) Review refugial areas and postglacial colonizations in the western Palearctic. In: Habel JC Assmann T (Eds) Relict Species: Phylogeography and Conservation Biology. Springer-Verlag, Berlin Heidelberg, 189–197. https://doi.org/10.1007/978-3-540-92160-8_11

[B11] Holderegger R Thiel-Egenter C (2009) A discussion of different types of glacial refugia used in mountain biogeography and phylogeography. Journal of Biogeography 36: 476–480. https://doi.org/10.1111/j.1365-2699.2008.02027.x

[B12] Holdhaus K (1954) Die Spuren der Eiszeit in der Tierwelt Europas. Universitätsverlag Wagner, Innsbruck, 493 pp.

[B13] Homburg K Drees C Gossner M M Rakosy L Vrezec A Assmann T (2013) Multiple glacial refugia of the low-dispersal ground beetle *Carabus irregularis*: Molecular data support predictions of species distribution models. Plos ONE 8: e61185. https://doi.org/10.1371/journal.pone.0061185 10.1371/journal.pone.0061185 PMC3617161 23593425

[B14] Jones P Lister D Osborn T Harpham C Salmon M Morice C (2012) Hemispheric and large-scale land-surface air temperature variations: An extensive revision and an update to 2010. Journal of Geophysical Research 117: 1–29. https://doi.org/10.1029/2011JD017139

[B15] Ladle R Jepson P Malhado A Jennings S Barua M (2011) The causes and biogeographical significance of species’ rediscovery. Frontiers of Biogeography 3: 111–118.

[B16] Ladle R Jepson P (2008) Toward a biocultural theory of avoided extinction. Conservation Letters 1: 111–118. https://doi.org/10.1111/j.1755-263X.2008.00016.x

[B17] Perrault G (1982) Le Genre *Leistus* (Froehlig) (Coleoptera - Carabidae - Nebriini) IV. Le sous-genre Pogonophorus Latreille: Division en group d’espèces. Bulletin mensuel de la Societé Linnéenne de Lyon 6: 169–175. https://doi.org/10.3406/linly.1982.10539

[B18] Pizzolotto R Albertini A Gobbi M Brandmayr P (2016) Habitat diversity analysis along an altitudinal sequence of alpine habitats: the Carabid beetle assemblages as a study model. Periodicum Biologorum 118: 241–254. https://doi.org/10.18054/pb.2016.118.3.3924

[B19] Pizzolotto R Gobbi M Brandmayr P (2014) Changes in ground beetle assemblages above and below the treeline of the Dolomites after almost 30 years (1980/2009). Ecology and evolution 4: 1284–1294. https://doi.org/10.1002/ece3.927 24834326 10.1002/ece3.927 PMC4020689

[B20] Scheffers B Yong D Harris J Giam X Sodhi N (2011) The World’s Rediscovered Species: Back from the Brink?. PLOS ONE 6: e22531. https://doi.org/10.1371/journal.pone.0022531 10.1371/journal.pone.0022531 PMC3144889 21818334

[B21] Szallies A Huber C (2014) Oreonebria (Marggia) bluemlisalpicola sp. nov., eine neue hochalpine Laufkäferart der nordwestlichen Schweizer Alpen (Coleoptera: Carabidae, Nebriinae). Contributions to Natural History 25: 5–21.

[B22] Vincent C Le Meur E Six D Funk M (2005) Solving the paradox of the end of the Little Ice Age in the Alps. Geophysical Research Letters 32: 1–4. https://doi.org/10.1029/2005GL022552

